# The Puzzle of Preimplantation Kidney Biopsy Decision-Making Process: The Pathologist Perspective

**DOI:** 10.3390/life14020254

**Published:** 2024-02-15

**Authors:** Albino Eccher, Jan Ulrich Becker, Fabio Pagni, Giorgio Cazzaniga, Mattia Rossi, Giovanni Gambaro, Vincenzo L’Imperio, Stefano Marletta

**Affiliations:** 1 Department of Medical and Sciences for Children and Adults, University of Modena and Reggio Emilia, University Hospital of Modena, 41100 Modena, Italy; 2 Institute of Pathology, University Hospital of Cologne, 50923 Cologne, Germany; janbecker@gmx.com; 3 Department of Medicine and Surgery, Pathology, IRCCS Fondazione San Gerardo dei Tintori, University of Milano-Bicocca, 20126 Milano, Italy; fabio.pagni@unimib.it (F.P.); giorgio9cazzaniga@gmail.com (G.C.); vincenzo.limperio@gmail.com (V.L.); 4 Division of Nephrology, Department of Medicine, University of Verona, 37129 Verona, Italy; mattia.rossi@univr.it (M.R.); giovanni.gambaro@univr.it (G.G.); 5 Department of Diagnostics and Public Health, Section of Pathology, University of Verona, 37129 Verona, Italy; stefano.marletta92@gmail.com; 6 Division of Pathology, Humanitas Istituto Clinico Catanese, 95045 Catania, Italy

**Keywords:** preimplantation kidney biopsy, pathologist, kidney transplant

## Abstract

Kidney transplantation is the best treatment for end-stage renal disease since it offers the greatest survival benefit compared to dialysis. The gap between the number of renal transplants performed and the number of patients awaiting renal transplants leads to a steadily increasing pressure on the scientific community. Kidney preimplantation biopsy is used as a component of the evaluation of organ quality before acceptance for transplantation. However, the reliability and predictive value of biopsy data are controversial. Most of the previously proposed predictive models were not associated with graft survival, but what has to be reaffirmed is that histologic examination of kidney tissue can provide an objective window on the state of the organ that cannot be deduced from clinical records and renal functional studies. The balance of evidence indicates that reliable decisions about donor suitability must be made based on the overall picture. This work discusses recent trends that can reduce diagnostic timing and variability among players in the decision-making process that lead to kidney transplants, from the pathologist’s perspective.

## 1. Context

**Figure 1:**
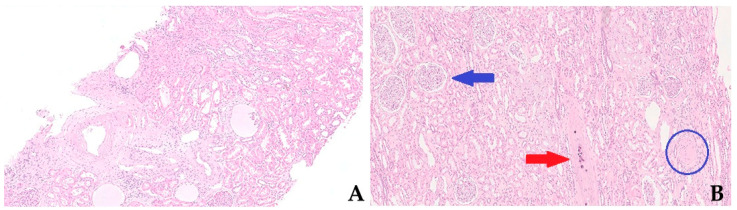
Histopathological picture of renal cortex in pre-implantation core-needle (**A**) and wedge biopsies (**B**), this latter highlighting several normal glomeruli (top left, blue arrow), a sclerotic glomerulus (bottom right, blue circle), and an arterial vessel with parietal calcifications (centre, red arrow).

Kidney procurement biopsies are a component of the process of organ quality evaluation before transplantation. Yet, the long series of models developed to cope with this issue, including a variety of clinical and histologic variables, failed to predict long-term graft survival correctly [1]. In reality, however, the question is far more complex, and from an anatomical–pathological point of view, this fiasco could be explained by the lack of standards in indication [1,2], sampling, and diagnostic definition [3,4]. There is no perfect correlation between tissue alterations and functional manifestations but, on the other hand, histologic examination of kidney tissue provides a unique window of the organ that cannot be inferred from clinical records and renal functional studies all along. Generally, the decision to use kidneys from deceased donors relies upon combinations of histological scoring and clinical parameters. Clinical and biochemical data are the most easily accessible for predicting graft quality at transplantation time. The Deceased Donor Score [5], the Donor Risk Score [6], and the Kidney Donor Risk Index [7] are the three most used scoring systems based on clinical and laboratory findings. These scores usually combine a variable number of clinical parameters, including the patient’s age, sex, and weight, with underlying pathological conditions causing death, like cardiovascular diseases and infections (CMV and HCV status), and the levels of biochemical markers, such as serum creatinine. Overall, these combined scores are supposed to forecast the glomerular filtration function of the graft before transplantation and have been consistently associated with graft survival [5,6]. Nevertheless, serum creatinine and blood urea nitrogen are inadequate renal function measurements, especially in patients with less than 50% reduction in glomerular filtrate [8]. On the other hand, preimplantation kidney biopsy is usually assessed with scores or slight modifications of them, and these comprise evaluation of the percentage of glomerulosclerosis (GS), the amount of interstitial fibrosis (IF), and tubular atrophy (TA), and arterial arteriosclerotic narrowing (Figure 1) [9,10]. The most used of these systems is the so-called semiquantitative Remuzzi–Karpinski score, which combines the aforementioned histological factors with other additional changes witnessing ischemia-related damage of the renal allografts [9].

**Figure 2:**
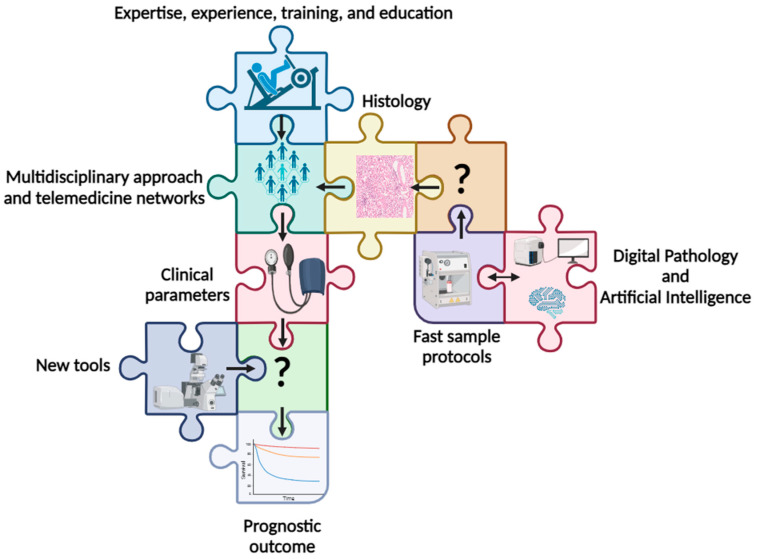
The interconnected players of the preimplantation kidney biopsy puzzle and their flow. Legend: “?”: still missing.

These considerations notwithstanding, the literature evidence confirms that histologic features are not strongly representative of organ status, and suboptimal scoring leads to suboptimal usage of organs [2]. Furthermore, there is a wide variability among pathologists, underlining that expertise, training, and interobserver variability constitute an essential issue. A lack of shared expertise among pathologists evaluating preimplantation kidney biopsy can be a significant problem for several reasons, primarily related to transplantation [11]. Likewise, the expertise of the pathologist is crucial for ensuring the success and safety of kidney transplantation procedures as it helps match the right donor kidney with the appropriate recipient and contributes to the ethical and efficient use of donor organs [12,13]. And just like a puzzle, the parts are put together into a challenge that must be addressed by requiring the data to be coherent and consistent with the safety of the care patients need. Given the intricate interplay between the pieces of the puzzle, it is certain that the issue still needs to be clearly outlined and that we probably do not know all of the key elements today. This review aims to outline the state of the art suggesting interventions for improving future practices in preimplantation kidney biopsy, including (i) expertise, experience, training, and education, (ii) digital pathology and artificial intelligence tools, (iii) multidisciplinary approach and professional networks among pathologists, nephrologists and surgeons powered by telemedicine, (iv) fast sample management protocols, (v) and new tools and machines (Figure 2).

## 2. Expertise, Experience, Training, and Education

Expertise and experience in transplant pathology are related concepts but have distinct meanings. Expertise represents a high level of understanding and competence that comes from formal education, training, and continuous learning. A pathologist expert in kidney transplantation would have a deep understanding of the principles, theories, and practical aspects of pathology related explicitly to kidney transplantation. Otherwise, experience refers to the practical knowledge and skills acquired through direct involvement in activities or situations. It involves the application of theoretical knowledge in real-world scenarios and the learning that comes from hands-on participation over time. A pathologist with experience in kidney transplant pathology has likely been actively involved in examining transplanted organs, analyzing pathology specimens, and interpreting results. This practical exposure contributes to a deeper understanding of the nuances and challenges that may arise in the field [14,15]. In the context of the preimplantation kidney biopsy, experience, and expertise, although unduly perceived as the same thing, usually go hand in hand and are considered as a value in the diagnostic process. Published evidence has highlighted that expertise and training greatly influence the final biopsy score and, therefore, greatly influence the potential discarding or inappropriate allocations [16]. Training and education in medicine are crucial for several reasons, playing a fundamental role in producing competent and skilled healthcare professionals. This is especially the case if it is a question of preimplantation kidney biopsy, specifically how often the on-call pathologists with no specific expertise are called to evaluate kidney biopsies for organ suitability. Moreover, timing is critical in transplant procurement, so detection of subtle alterations potentially influencing the function of the graft in the recipient, like thrombotic microangiopathy changes, tubular necrosis, or grading diabetic damage, can be very challenging.

Simulation-based learning (SBL) has been claimed as an effective way to improve safety and quality in healthcare by replicating real-world situations [17]. SBL provides a controlled and immersive environment that allows professionals to enhance their skills, knowledge, and decision-making abilities. The key reasons why SBL is a useful tool are skill development and standardized assessment (favoring pathologists to repeatedly practice and refine specific tasks, such as preimplantation kidney scoring), scenario replication (this enables pathologists to experience and learn how to handle complex cases or situations that they may encounter infrequently in routine practice), decision-making skills (this is especially important in high-pressure situations like transplant practice where quick and accurate decisions can be critical for patient outcomes) and continuous learning (SBL provides a platform for healthcare professionals to stay updated and continuously improve their skills). Medical education and training hone competence and critical thinking skills, and researchers have detailed the benefit of simulation responses to unanticipated needs as they present, which is actually not possible in current patient care situations. Data from pre- and post-simulation feedback demonstrated significant improvements in technical knowledge, diagnostic awareness, and confidence in clinical management following simulation exposure. Ultimately, SBL has been demonstrated as an effective way for upskilling practitioners, so it can, therefore, be a means for improving the clinical practice of preimplantation biopsy in a safe and simulated environment [18].

## 3. Digital Pathology and Artificial Intelligence

**Figure 3:**
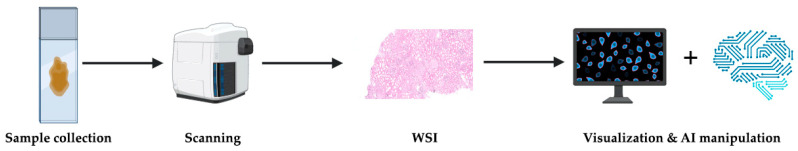
The digital pathology workflow: conventional histological slides are scanned to whole slide imaging (WSI) and then visualized on a computer monitor where they can be freely manipulated (rotated, zoomed in and out, etc…) by pathologists, eventually with the support of artificial intelligence (AI) tools.

Digital pathology (DP) plays a key role in speeding up the progression of healthcare, and the potential benefits of adopting digital technologies have been solidly proven (Figure 3) [19]. Academia is making a great effort to implement DP into clinical practice [20]; however, despite great strides in recent years, the employment of DP remains limited to only a few pathology laboratories [21]. The main causes that hindered the spread of digital pathology included costs of implementation, as the initial costs associated with implementing digital pathology systems, including the purchase of scanners, software, and the required IT infrastructure, can be substantial. Although the savings benefits have been established [22], many healthcare institutions, especially smaller ones, may find it challenging to allocate resources for such investments. Integrating digital pathology systems with existing infrastructure can be complex, and compatibility issues, data migration, and workflow integration have slowed down the adoption process. Furthermore, despite the availability of guidelines and recommendations for regulatory and standardization purposes that comprehensively cover every step of the implementation of the digital workflow [23,24], stressing out the value of interoperability, automation, and tracking of the whole process, institutions have been not confident in the adoption and implementation of these technologies. But perhaps the most important barrier to the adoption of DP has been cultural, with pathologists and other healthcare professionals accustomed to traditional methods and reluctant to embrace new technologies. Despite these challenges, the adoption of digital pathology is increasing as the technology matures, costs decrease, and more evidence and awareness supporting its benefits emerge [25].

Transplantation pathology is a highly specialized field and most professionals do not have enough expertise to handle critical practice needs as the limited number of cases hinders the development of necessary skills. DP has the potential to significantly impact the diagnostic organizational model in transplant pathology as it allows the on-call pathologists to employ intraoperative consultation and rapidly gain an expert second opinion [26,27,28]. Other advantages of DP application in transplant pathology include enhanced collaborations, quality control, data management and analysis, and workflow efficiency. Specifically, there is the consolidated literature evidence certifying the role of DP in speed and ease in the diagnostic management of preimplantation kidney renal biopsy [29,30]. The introduction of DP in clinical trials, research, and practice has triggered the application of artificial intelligence (AI) to histopathology, with the development of novel machine learning models for tissue interrogation and discovery [31,32]. In the current era of precision medicine, it is necessary to recognize and quantify histopathological kidney features in an objective way in order to discover correlations between these findings with clinical parameters and transplant outcome data. The literature evidence on the application of AI to preimplantation biopsies is still scarce and more focused on more straightforward tasks, like counting normal and sclerotic glomeruli, vascular structures, and quantifying interstitial fibrosis [33,34]. Among the pioneer studies published on this topic, for instance, the RENFAST (Rapid EvaluatioN of Fibrosis And vesselS Thickness) is an AI-based algorithm developed to recognize interstitial fibrosis and changes in vascular walls. This tool was trained on a series of 300 renal biopsies and achieved better results than previously tested software and conventional light microscopy. Furthermore, it performed much faster than the evaluation of glass slides, with a 2 min average time of examination compared to 20 min with classic methods [35]. Several issues are indeed to be resolved for definitely improving the performances of such algorithm, including the addition of tubular and glomerular analysis and proper distinction between cortical and medullary renal tissue; however, such software could represent the first promising example of innovative informatics solutions alleviating transplantation pathologists routine workload. Such AI models would particularly suit the evaluation of biopsies from “marginal” extended criteria donors (ECDs), providing transplantation physicians with fast and reliable estimation of clinically relevant parameters [36].

There are several artificial intelligence models used for different tasks, and they can be broadly categorized into three main types: rule-based systems, machine learning models (ML), and deep learning models (DL). ML models learn patterns and make predictions or decisions without being explicitly programmed. They improve their performance as they are exposed to more data. DL is a subset of ML that involves neural networks with multiple layers (deep neural networks). These models can automatically learn hierarchical representations of data. The choice of an AI model depends on the specific task, the type of data available, and the desired outcomes. Some tasks may benefit from a combination of different models or techniques, known as ensemble methods. Models based on ML have been introduced in solid organs transplantation, whose prognosis depends on a complex, multidimensional, and nonlinear relationship between variables pertaining to the donor, the recipient, and the surgical procedure [37,38]. Given the importance of assessing preimplantation kidney histopathology with utmost accuracy and precision, one of the main goals of AI is to reduce interobserver variability. Low inter-pathologist agreement can have an important impact on the entire transplant’s management. Notably, this kind of discordance is not limited to kidney preimplantation biopsy assessment. Still, it is ubiquitous in any classification based on morphology, ranging, for example, from the Banff criteria for transplant rejection to other pathology settings where human skill and experience can represent an added value but also represent a limit [39]. However, it is worth noting that we probably still do not exactly know the most essential histologic features related to the graft outcome and their cut-off. In this context, we can understand the importance of staying focused on measurable, objective features, such as the number and type of glomeruli and vessels, excluding the questionable and poorly reproducible ones. Projects developing AI tools need to involve transplant professionals to strengthen trust and facilitate a smooth translation to the clinic. From a pathologist’s perspective, the key to unlocking trust in AI will be designing models optimized for intuitive and friendly human–AI interactions and ensuring that, where judgment is required to resolve grey zones, the tool’s working mode is controllable and understandable to the human observer. This is why the first models should be simple and focus on objective measurements that can be easily verified by the “human leader” of the diagnostic process.

The last challenge would be human–AI integrating clinical tabulations and whole slide images into flexible machine learning models or an ensemble model with variable inputs, specifically designed to improve clinical decision making and patient outcomes.

## 4. Multidisciplinary Approach and Telemedicine Networks

Different clinical–pathological models have been proposed and tested for assessing donor kidney quality [40]. It is hard to establish the real significance of the preimplantation histologic features, as this has often led to the suboptimal use of the organs, with a limited predictive value on their function and longevity after transplantation. A multidisciplinary approach in the preimplantation diagnostic context is crucial because it leverages the expertise of various healthcare professionals to provide comprehensive, well-coordinated care, reduces errors, enhances transplant outcomes, and makes more efficient use of resources. This approach recognizes that preimplantation kidney assessment is complex in every aspect, requiring diverse perspectives and skills to address the various frameworks. The aim is to promote the design and development of models focused on the transplant outcome indicators and to develop evidence-based guidance on the standardization and clinical utility of preimplantation kidney biopsy for the evaluation of grafts. To this end, an international multidisciplinary panel made up of leading experts in pathology, nephrology, and transplant surgery took stock of the available literature evidence during the Transplantation Learning Journey (TLJ), coordinated by the European Society for Organ Transplantation (ESOT) in Prague (13–15 November 2022). The main aim was to develop methodologically solid, consensus-based guidance on clinical practice to establish guidelines on crucial aspects of preimplantation histopathology’s role in the graft assessment process. A detailed, systematic literature review of the topic was performed to provide definitive evidence and expert opinion. The methodology allowed us to reach a solid consensus on various technical issues regarding the preimplantation kidney biopsy, and, at the moment, it represents the first attempt in Europe to standardize procedures with a multidisciplinary approach [3]. Telemedicine networks play a crucial role by leveraging technology to facilitate real-time exchange of views among the professionals (pathologists, nephrologists, and surgeons) involved in the decision-making process of kidney transplants [41].

Examples of connected telepathology units are well known in the literature, such as the intraoperative telepathology service in South Tyrol [42] and the local network in Eastern Sicily [43]. Although such validated models have focused on other fields above transplantation, like frozen section and routine surgical pathology specimens, the evaluation of pre-implantation kidney biopsies would certainly benefit from similar systems. This would be particularly valuable for donor procurement sites located in remote or underserved areas, providing them with real-time support with access to transplant expertise [27]. However, developing standards of practice and coordinating diagnostic procedures with qualified on-call pathologists takes planning, time, and money. Some organizations, such as Eurotransplant, have developed a wide expertise in working across national boundaries. Likewise, areas with consolidated experience in transplantation pathology are about to embrace projects of shared fully digital workflows. In this context, for instance, the Veneto region in northern Italy is about to undergo a total digital transformation of pathology laboratories in the very near future. It will represent a frontrunner in the field. The costs of developing, implementing, and maintaining these networks require human resources, tools, and money. However, different cost-saving strategies could impact the complex changing economic benefits of transplantation [44]. The reassurance that every professional involved in the transplant process would have in gaining access to a telemedicine network would favor sharing expertise and reinforce mutual confidence. Therefore, ultimately, a telemedicine network planned to manage the entire preimplantation kidney evidence can ensure the potential to increase the organ donor pool and improve the allocation and number of transplants [30].

## 5. Fast Sample Management Protocols and Stains

Turn-around time (TAT) is a compelling issue in preimplantation kidney biopsy: the faster the diagnosis is rendered, the greater the graft benefits. Indeed, the overnight processing procedure of routine pathology specimens does not fit with the urgent needs of transplantation. Thus, preimplantation kidney biopsies commonly undergo intraoperative frozen-section examination in most transplantation units. Despite being high-speed, slides obtained with such a technique are often effaced by significant artifacts, which may affect their interpretation. For instance, subtle morphological changes in the amount of arterial hyalinosis and deposition of mesangial matrix may be underestimated by frozen-section and could be so easily overlooked by an inexperienced pathologist [4]. Therefore, the Banff working group warranted to investigate rapid formalin-fixation and paraffin-embedding protocols for histological evaluation of the preimplantation renal biopsies [9]. In this view, modifications of routine methods have led to the development of rapid tissue processing procedures, allowing pathologists to be provided with hematoxylin and eosin (H&E) stained slides in about 4 h. In the last thirty years, microwave tissue processing has gained popularity, as witnessed by the commercial development and release of ovens designed to guarantee homogeneous and quick processing under accurately controlled tissue temperatures and conditions. Namely, these procedures rely on microwaves to rapidly dehydrate previously fixed tissue in only one step by heating the reagent alcohol solution just below its boiling point. In addition, paraffin impregnation occurs at a higher temperature than conventional processing, further speeding the process up [45]. It is also worth mentioning that, despite its widespread acceptance being linked to immunohistochemistry, originally microwave technology had been first employed for tissue processing in routine morphological preparation.

In the last decades, several studies have reported comparable results on the diagnostic ability of conventional and microwave tissue processing [45,46]. Being a labor-intensive method, microwaves have been primarily applied to the processing of small biopsies taken from patients for whom the diagnosis is crucial to guide life-saving treatments. Among these, for instance, should be accounted as gastrointestinal, bronchial, and hepatic samples from immunodeficient subjects or patients affected by rapidly growing neoplasms such as lung small cell neuroendocrine carcinomas. The proficient application of microwaves to renal biopsy processing has been known for years, with various investigators reporting no substantial change in renal architecture and cellular morphology compared to conventional techniques [47]. Therefore, current evidence advocates for its wider adoption in preimplantation procedures, as it may represent a safe and feasible way to shorten TAT without a demonstrable reduction in the samples’ quality.

Finally, it is also worth mentioning that, alongside H&E, special histochemical stains may further support physicians in evaluating specific morphological features of preimplantation renal biopsies [48]. For instance, Periodic acid-Schiff (PAS) can underline glomerular alterations such as GS and vascular changes: the PAS stain eases the recognition of arteriolar hyalinosis as well as the increase in the mesangial matrix. Moreover, in comparison with H&E, the PAS staining, by highlighting the tubular basement membranes, supports pathologists for a more proper quantification of the amount of the renal interstitium. As for this latter, colorations for stromal tissue like Masson Trichrome may help pathologists in the estimation of fibrotic modifications of interstitial tissue. Thus, broad employment of such affordable ancillary is advisable, as they may ultimately prompt vital information influencing the overall graft’s management decision-making process.

## 6. New Tools

Technological development plays a crucial role in the advancement of healthcare and in the last few years, new emerging tools have had a significant impact in the field of transplantation. Perfusion machines provide a method to preserve and optimize the condition of organs before transplantation. Historically, renal grafts recovered from deceased donors have been maintained using ice, also called static cold storage (SCS). Since the 1960s, different machine-based methods have been developed that not only allow kidneys to be perfused in a much more physiological way but which also let physicians constantly evaluate parameters related to the organs’ function and, ultimately, improve the grafts’ outcomes. This technique involves pumping a specialized preservation solution through the organ’s blood vessels to maintain its viability and function. Different kinds of liquids are available nowadays as long as pulsatile systems and temperature-regulating machines keep the harvested organs in solutions closely resembling its physiological conditions. Some advantages of perfusion machines are: extended preservation time (this is essential for organs that need to be transported over longer distances or when there is a delay between organ retrieval and transplantation), improved organ viability (perfusion with a preservation solution helps to maintain optimal oxygenation and nutrient supply to the organ), organ evaluation/assessment (functional parameters can be monitored during perfusion), treatment of ischemia-reperfusion injury (allowing for interventions during the perfusion process to mitigate ischemia-reperfusion injury, enhancing the organ’s post-transplant performance), and improving transplantation from marginal donors (improving the condition of these organs and making them more suitable for transplantation) [49,50,51]. In detail, compared to SCS, machine-perfused kidneys have revealed significantly lower rates of delayed graft function (DGF) and, overall, reduced transplant-related costs. This is especially true when employing the hypothermic machine perfusion (HMP) technique, which consists of storing grafts at 4° C prior to transplantation [52]. Moreover, different studies have stressed putative associations between graft survival and parameters measured during its perfusion before transplantation, including perfusion pressure, perfusion flow, and renovascular resistance (RR), among others. The latter is the ratio between vascular pressure and flow during machine perfusion. Evidence supports RR as a parameter correlated with the development of DGF and graft survival one year after transplantation, especially in patients benefitting from organs from ECDs [53]. However, parameters measured by machine perfusion techniques are not enough to accurately predict graft outcomes all along. Thus, integration with other clinical and laboratory quickly accessible findings in the near future may, at least partially, sort this problem out [54]. In this view, perfusion machines allow for the eventual planning of the biopsy for the histological evaluation of the kidney to be transplanted, offering a time window for the multidisciplinary discussion of the findings, a second diagnostic opinion, and the application of artificial intelligence algorithms.

In this view, novel imaging techniques coupled with machine-perfusion technologies offer the opportunity to deeply investigate grafts’ function before transplantation in a non-invasive way. For instance, a recent study applied magnetic resonance imaging (MRI) to kidneys during ex vivo normothermic machine perfusion (35–37 °C). The investigators showed how this technique may work as a reliable method for assessing both renal metabolism and physiology, providing clinicians with a realistic picture of critical biological parameters, including microenvironmental oxygen availability, local perfusion flow, and drug distribution, among others [55]. Similarly, another work aimed to estimate the oxidative metabolism of renal grafts during ex vivo organ perfusion by a 3-Tesla MRI scanner was able to detect the oxygen-17 isomer [56]. The authors elegantly recorded the levels of oxidative metabolism in the organ, with higher rates in the renal cortex and lower in the medulla, likely reflecting its functional quality. To note, MRI techniques have been employed for years to indirectly study the functionality of renal tissue. On this tissue, brilliant articles showed the ability of ^31^P MRI spectroscopy during the cold ischemia period to forecast the likelihood of developing acute tubular necrosis immediately after transplantation [57].

In the last few decades, transplant professionals have been struggling to identify circulating or urinary biomarkers allowing them to predict the graft’s function in a non-invasive way compared to histological examination. Several candidates have been variably claimed as potentially able to reflect molecular alterations of the selected organs and therefore forecast acute and chronic post-transplant complications. As for the formers, encouraging results have been mainly linked to the serum and urinary levels of different inflammatory-related molecules. For instance, several studies have shown that patients with elevated pre-transplant plasmatic levels of the soluble forms of CD30, CXCL10, and endotrophin (a portion of collagene type IV) carry a higher likelihood of developing acute graft rejection [58,59]. Similarly, the urinary rates of neutrophil gelatinase-associated lipocalin have been linked to the incidence and severity of delayed graft function [58]. Like, other easily assessable biomarkers have been identified as useful tools to detect chronic graft damage before its histological evidence or clinical manifestation. Speaking of this, pre-transplant serum levels of NT-proBNP were independently associated with all-cause mortality in a wide series of kidney-transplanted patients more than 10 years after the study [60]. In summary, ongoing evidence advocates for the seeking of specific pre-transplant predictive biomarkers which, supporting subsequent histological exam of the harvested organs, will help to target the recipient’s clinical management by identifying the graft’s probability of developing acute and chronic damages.

The fluorescence confocal microscope (FCM) is a specialized type of microscope that uses a scanning method to produce high-resolution images of biological samples. FCM rapidly creates images of fresh samples with a resolution comparable to conventional light microscopy. FCM can have a crucial role in preimplantation kidney biopsy, allowing a fast and real-time analysis of the tissue at different depths. Confocal images well correlated with the corresponding conventional histological pictures, both in normal tissue and chronic lesions (GS, IT, and TA) [61,62]. FCM can be a turning point as it is a material-sparing method that provides rapid diagnostics feedback, maintaining its quality for further examinations. This can lead to faster and safer therapeutic choices in the management of donors and recipients, increasing the number and the safety of transplants [63].

## 7. Conclusions

Connecting the pieces of the preimplantation kidney biopsy puzzle is the priority for the scientific transplant community. Some evidence is in contrast and there are actually many unsolved challenges. Still, emerging novelties hopefully will help to figure these issues out and merge the knowledge of various specialties, such efforts are perhaps witnessed more than ever by a multidisciplinary, methodologically solid, perfectly reproducible, and innovative approach.

## Data Availability

Not applicable.
